# Awareness and acceptability of monkeypox vaccine in men who have sex with men

**DOI:** 10.55730/1300-0144.5679

**Published:** 2023-08-11

**Authors:** Arda KARAPINAR, Damla AKDAĞ, Deniz GÖKENGİN

**Affiliations:** 1 Red Ribbon İstanbul Association, İstanbul, Turkiye; 2 Department of Infectious Diseases and Clinical Microbiology, Başakşehir Çam Sakura City Hospital, İstanbul, Turkiye; 3 Department of Infectious Diseases and Clinical Microbiology, Faculty of Medicine, Ege University, İzmir, Turkiye; 4 Ege University HIV/AIDS Research and Practice Centre, İzmir, Turkiye

**Keywords:** HIV, monkeypox vaccine, men who have sex with men, prophylaxis

## Abstract

**Background/aim:**

To determine the knowledge about and acceptance level of monkeypox vaccine in men who have sex with men (MSM).

**Materials and methods:**

A 14-item questionnaire, developed by the European Centers for Disease Control (ECDC), was presented online to MSM, aged ≥18 years old, via smartphone applications (Grindr and Hornet), between June 30th and August 12th, 2022.

**Results:**

Of the 737 participants who completed the survey, 678 were born in Türkiye and 59 were migrants/visitors born in different countries. All of the participants were living in Türkiye. The median age was 31 (range 18–68) years. Overall, 21.9% were HIV-positive, 94.9% were using antiretroviral therapy (ART), 3.9% reported using preexposure prophylaxis (PrEP), 9.9% had been diagnosed with a sexually transmitted infection (STI) in the past 12 months, and 10.1% reported using chemicals during sexual intercourse in the past three months.

Participants aged 45–54 were significantly more concerned about being treated differently due to monkeypox than those in the other age groups (p = 0.038). Compared to the participants who were HIV-negative, those who were HIV-positive were significantly more worried about acquiring monkeypox (34.1% were very worried and 43.6% were worried, p = 0.033), were more likely to definitely or probably get vaccinated if offered (67.6% and 80.6%, respectively, p = 0.002), and were more concerned about being exposed to different attitudes due to monkeypox (37.0% and 53.3%, respectively, p < 0.01). Among those using ART, 82.3% and 50.0% reported that they would definitely or probably get vaccinated if offered, respectively (p = 0.046). There were no significant differences between groups for the remaining parameters.

**Conclusion:**

Despite the low level of knowledge about monkeypox, the majority of the participants reported that they believed in the vaccine’s efficacy. Those who were HIV-positive were particularly more concerned about monkeypox and were more willing to protect themselves compared to those who were HIV-negative.

## 1. Introduction

Monkeypox is a viral zoonotic infectious disease that was first identified in humans in 1970 [1] and remained endemic in Central and West African countries probably due to their active global smallpox vaccination programs. Sporadic cases occurring in other regions were associated with travel and human-to-human transmission was considered insignificant [2, 3]. However, following the discontinuation of routine vaccination after the eradication of smallpox, the immunity level of the populations decreased, causing monkeypox to become a global public health issue. Over the years, outbreaks and sporadic cases have been reported in regions outside of Africa^1^ [4]. Then, in the first half of 2022, there was a sudden increase in the number of monkeypox cases in nonendemic European countries and the United States of America (USA). The first local cases of monkeypox were confirmed in the United Kingdom (UK), followed by many other European countries^2^,^3^. Following the official reporting of more than 5000 cases from over 50 countries in 5 regions by May 2022, the World Health Organization (WHO) declared monkeypox as a “Public Health Emergency of International Concern” on July 23rd, 2022^4^. The first confirmed case in Türkiye was reported on June 30th, 2022^5^.

According to data from the WHO and the Centers for Disease Control and Prevention (CDC), as of April 12th, 2023, 86,956 people worldwide have been infected with monkeypox, and 119 people have died during the current outbreak that began in May 2022^6^. Males represented the vast majority of infected patients (approximately 99%), and male-to-male sexual intercourse was the primary method of transmission, according to reports [5–11]. Transmission has also been reported via heterosexual contact [12–18], nonsexual close contact of children with their caregivers [19–21], percutaneous injury from a needle/instrument contaminated by an infected skin lesion [22–25], piercing and tattooing [26–28], and occupational exposure without proper personal protective equipment [29, 30]. Although it remains unclear whether human immunodeficiency virus (HIV) infection affects the risk of acquiring monkeypox, cohort data from the 2022 outbreak showed that, among those infected with monkeypox, the percentage of people who were HIV-positive varied between 36% and 42% [6, 9, 31].

Immunization by vaccines is a critical strategy in the fight against infectious diseases, especially during outbreaks. To achieve the desired coverage, a public health strategy that ensures the dissemination of correct information to the public and encourages vaccination should be adopted. However, vaccine hesitancy has become a growing trend in recent years, resulting in a severe decline in vaccine acceptance. Failure to achieve the desired immunity target during the COVID-19 pandemic and the recent increase in measles cases in Türkiye both support this trend [32]. A similar scenario is anticipated with the monkeypox outbreak due to a similar vaccine reluctance. Lack of knowledge and misinformation about the disease and/or vaccine, fear of possible adverse effects, lack of trust in medical professionals or the healthcare system, and anxiety about stigmatization all negatively impact vaccination rates. Similarly, sociodemographic characteristics such as age, gender, and area of residence are also determinants of vaccine acceptance [5, 33].

In controlling the monkeypox outbreak, along with preventive and education initiatives, mass immunization by vaccination is the most crucial approach for reestablishing herd immunity. While trials are still ongoing to produce effective and safe vaccines, licensed vaccines are already being used for high-risk populations in countries such as the USA, Canada, and the UK [5]. Considering that the virus is transmitted through close contact within certain communities, vaccinating men who have sex with men (MSM), as a defined high-risk group, is considered the most rational approach to protect individuals from the disease and prevent it from spreading. The barriers to vaccine acceptance in target populations must be identified and removed to increase coverage.

This study aimed to determine the knowledge about and acceptance level of monkeypox vaccine among MSM living in Türkiye.

## 2. Materials and methods

This cross-sectional survey study was designed and conducted by the Ege University, Faculty of Medicine, Department of Infectious Diseases and Clinical Microbiology. A 14-item questionnaire (Table 1) was used to assess awareness and acceptance of the monkeypox vaccine among MSM, which was developed by the European Centers for Disease Control and Prevention (ECDC) and translated into 25 languages, including Turkish [34]. The survey was administered online via smartphone-based gay dating apps (Grindr and Hornet) to MSM aged ≥18 years living in Europe, between June 30th and August 12th, 2022. The survey was completed by 32,902 respondents, and of those, 737 who reported to be living in Türkiye were included in the analysis.

The statistical analyses were performed using the IBM SPSS Statistics for Windows 22.0 (IBM Corp., Armonk, NY, USA). The categorical variables were expressed as frequencies and percentages, while the continuous variables were expressed as the mean ± standard deviation and median (minimum–maximum). Differences between the groups (HIV-positive vs. HIV-negative/unknown status; on ART vs. not on ART) for the categorical independent variables were assessed using the chi- squared and Fisher exact tests. The first type margin of error was determined as α: 0.05, and p < 0.05 was considered statistically significant.

## 3. Results

Of the 737 participants who completed the survey, 91.9% (678/737) were born in Türkiye and 8.0% (59/737) were born in another country; the median age was 31 (range: 18–68) years; 21.9% (156/713) were HIV-positive, and 94.9% (148/156) of those who were HIV-positive reported using antiretroviral therapy (ART). Of the respondents, 3.9% (22/569) reported using preexposure prophylaxis (PrEP) to prevent HIV infection in the past 3 months, 16.0% (117/729) reported being diagnosed with a sexually transmitted infection (STI) in the past 12 months, and 10.1% (73/723) reported using a chemical substance (mephedrone, gamma-hydroxybutyrate/gamma-butyrolactone, ketamine, or crystal methamphetamine) during sexual intercourse in the past 3 months. Of the participants, 2 (0.27%; 2/734) reported being infected with monkeypox, and 13 (1.77%; 13/734) reported knowing someone who was infected with monkeypox.

Of the respondents, 45.5% (333/731) and 24.7% (181/731) reported that they would accept and probably would accept vaccination if offered, respectively, with an overall acceptance rate of 70.3%. However, 10.53% (77/731) of the respondents were against vaccination; 4.51% (44/731) reported that they probably would not accept vaccination and 6.01% (33/731) stated that they definitely would not. Responses to the other survey questions are presented in Table 2.

In the subgroup analyses, responses to questions related to the level of knowledge about the vaccine efficacy, the severity of monkeypox disease, and concerns about acquiring the disease did not differ significantly between the age groups. However, participants in the 45–54 age group were significantly more concerned about being treated differently due to monkeypox compared to those in the other age groups (p = 0.038).

There was a significant difference in vaccine acceptance rates between the participants who thought monkeypox was very severe or severe and those who answered moderate, mild, or very mild. Those who thought the disease was very severe or severe were more likely to state that they would definitely or probably get vaccinated if offered (80.1% and 68.3%, respectively; p = 0.005) compared to the other participants. Regarding the impact of stigma on vaccine acceptance, a significantly higher number of participants who believed they would be treated differently due to monkeypox were willing to be vaccinated compared to those who answered no or do not know (83.6% and 61.2%, respectively; p < 0.01).

When compared to the participants who were HIV-positive, those who were HIV-negative had a significantly higher rate of being very worried or worried about acquiring monkeypox (43.6% and 34.1%, respectively; p = 0.033), reporting that they would definitely or probably get vaccinated if offered (80.6% and 67.6%, respectively; p = 0.002), and believing they would be treated differently due to monkeypox (53.3% and 37.0%, respectively; p < 0.01). When analyzed according to ART use, the percentage of those who stated they would definitely or probably get vaccinated if offered was significantly higher among those on ART than those who were not (82.3% and 50.0%, respectively; p = 0.046) (Table 3). No statistically significant differences were observed between the groups for the remaining parameters.

## 4. Discussion

This study has shown that despite the low number of monkeypox cases in Türkiye and the unavailability of the vaccine, the acceptance rate for monkeypox vaccine is high, particularly among people who are HIV-positive.

The study group, which consisted of users of online dating apps, was expected to exhibit higher levels of risky sexual behavior than those who do not use the app or the general population and consequently, to have a higher level of knowledge about monkeypox [35]. However, the level of knowledge in the survey was much lower than expected. Similarly, the level of knowledge among people who were HIV-positive, using PrEP, had a history of STI, or chemical sex experience was also low. Despite the low level of knowledge about the disease, the majority of the participants believed that the vaccine would be protective, and the reported vaccine acceptance rate was quite high, at 70.3%. While this rate is somewhat lower than the 82% defined for all European countries in the ECDC study, it is close to the 66.2% average reported for Central European countries [34]. A systematic review and metaanalysis published in 2022, which included 11 studies conducted in 10 different countries and 8045 individuals, reported an overall 56% acceptance rate for monkeypox vaccine; in the subgroup analyses, the acceptance rate was lower (50.0%) in Asian countries and higher (70.0%) in Europe [5]. In the population-based subgroup analyses of the same study, while the vaccine acceptance of the general population was quite low (43.0%; 95% CI: 35.0%–50.0%), it was almost 2-fold higher in lesbian, gay, bisexual, transgender, intersex (LGBTI) communities (84.0%; 95% CI: 83.0%–86.0%). These results suggest that social and cultural factors, relationships within a community, and information exchange might have a critical role in vaccine acceptability. However, the vaccine acceptance rates based on our study results were relatively lower compared to those for LGBTI population-based studies. This may be attributed to the fact that the number of confirmed cases in Türkiye has not reached alarming levels in contrast to other European countries, and the unavailability of the vaccine^7^. It is clear that awareness-raising interventions among MSM and other key populations about monkeypox and vaccines has not reached the desired level in Türkiye, and collaboration between public health authorities and community-based organizations (CBOs) are required to address this issue.

Monkeypox is not clearly linked to HIV infection, and there is no evidence that being infected with HIV increases the risk of acquiring the infection; the risk of severe monkeypox was reported to be higher only in individuals with low CD4+ T lymphocyte counts [36]. However, respondents who were HIV-positive were significantly more concerned about the risk of acquiring monkeypox and being exposed to discrimination if they did. This may be due to the higher incidence of HIV-positive individuals among those infected during the outbreak^8^,^9^, as well as the fear of health deterioration and stigma caused by HIV itself. However, these concerns were reported to have several positive outcomes, such as increasing vaccine acceptance and reducing risky sexual behaviors. Many studies have shown that the perception of monkeypox as a severe disease or that it would cause discrimination increases vaccine acceptance [34, 37, 38]. Similarly, thisthe present study found that individuals who expressed similar concerns had a significantly higher vaccine acceptance rate. Parallel to these concerns, the vaccine acceptance rate among the participants who were HIV-positive was significantly higher than that of the other participants. In an online survey of gay, bisexual, and other MSM in the USA, 48% of participants reported reducing the number of sexual partners, 50% reported reducing the frequency of 1-time sexual encounters, and 50% reported reducing sexual encounters with partners met through dating apps or in sex venues due to the monkeypox outbreak^10^.

Although participants who were HIV-positive expressed greater worry about both monkeypox and its consequences, as well as being stigmatized if they became infected, the scenario was different for ART users. While the level of concern among ART users was similar to that of the other participants, their vaccine acceptance rate was significantly higher. This may be due to the positive effects of being within the healthcare system and the perception of being safe with ART controlling HIV infection. In Türkiye, the close relationship between doctors and patients during the follow-up of HIV infection leads to an increase in health literacy and knowledge about diseases. In addition, patients generally trust healthcare providers, treatments, and the functionality of health policies, which may have increased vaccine acceptance without increasing concern. However, vaccine acceptance levels were not as high as expected among PrEP users or those with a history of STI. Effective use of social media and mass media to disseminate accurate information, increasing health literacy in the community, establishing an environment where individuals trust health authorities and healthcare providers, particularly in key communities at high risk of acquiring monkeypox, are critical for eliminating negative attitudes and approaches to vaccines.

This study is the first and only survey conducted in Türkiye to comprehensively demonstrate the awareness and acceptance of monkeypox and the vaccine among MSM. However, there were several limitations to the study:

1. Only MSM who use dating apps were included in the survey, and these individuals may engage in higher-risk behaviors compared to those who do not use such apps. [39].
2. Those who perceive a higher risk of acquiring the disease may be more willing to accept vaccination, which could lead to higher acceptance rates than expected.
3. The survey results may not reflect the attitudes of individuals with low digital literacy.
4. The results were based on subjective responses from the participants, which may have led to biased answers.
5. The impact of socioeconomic factors such as race, religion, and immigration status on vaccine acceptance was not analyzed.

## 5. Conclusion

Although the knowledge level about monkeypox is low in Türkiye, people who are HIV-positive are greatly concerned about acquiring monkeypox and are willing to protect themselves. Providing evidence-based information in a user-friendly format is critical for an effective monkeypox control program. Especially for MSM infected with HIV who do not use ART or have a lower risk perception of infection and a high level of hesitancy regarding vaccination, a better connection with CBOs and healthcare practitioners seems essential. The health-seeking behaviors of these groups may significantly differ from other MSM subgroups, and therefore, efforts to promote and improve health should be adapted to meet their needs.

## Figures and Tables

**Table 1:** Survey questions.

Questions
1.* In which country do you live?
2.* Where were you born?
3.* How old are you?
4. Are you HIV-positive?
5. Have you taken preexposure prophylaxis (PrEP) for HIV in the last 3 months?
6. In the last 12 months, have you been diagnosed with any other sexually transmitted infections?
7. Have you used mephedrone, GHB/GBL, ketamine, or crystal methamphetamine during sex with other sexual partners within the last 3 months?
8. How much do you agree with the following statement: “Vaccines protect us from many diseases”?
9. How severe a disease do you think monkeypox is?
10. Have you or someone you know been diagnosed with monkeypox?
11. How do you feel about your risk of acquiring monkeypox?
12. If the vaccine for monkeypox is offered to you, will you get vaccinated?
13. Where will you prefer to be vaccinated?
14. Have you been worried about being treated differently due to monkeypox?

Questions marked with an asterisk (*) were mandatory in the survey.

**Table 2:** Responses of the participants to the survey questions.

How much do you agree with the following statement: “Vaccines protect us from many diseases”? (Number of respondents: 735)	
Strongly agree	47.2% (347)
Slightly agree	23.0% (169)
Neither agree nor disagree	7.5% (55)
Slightly disagree	8.8% (65)
Strongly disagree	10.7% (79)
I do not know	2.7% (20)
How severe a disease do you think monkeypox is? (Number of respondents: 737)	
Very severe	22.4% (165)
Severe	0.1% (1)
Moderately severe	33.8% (249)
Slightly severe	15.1% (111)
Not severe	4.5% (33)
I do not know	24.2% (178)
How do you feel about your risk of acquiring monkeypox? (Number of respondents: 737)	
Very worried	11.7% (86)
Worried	22.5% (166)
Moderately worried	20.9% (154)
Slightly worried	26.3% (194)
Not worried	12.2% (90)
I do not know	6.4% (47)
If the vaccine for monkeypox is offered to you, will you get vaccinated? (Number of respondents: 736)	
I will get vaccinated	45.2% (333)
Probably yes	24.6% (181)
Not sure	19.0% (140)
Probably not	6.0% (44)
I will not get vaccinated	4.5% (33)
I do not want to answer	0.7% (5)
Where would you prefer to be vaccinated? (Number of respondents: 698)	
At an STI clinic	8.6% (60)
With my general practitioner	14.0% (98)
At a community-based center	1.1% (8)
At a vaccination program center	27.5% (192)
It does not matter	42.0% (293)
I do not want to answer	6.7% (47)
Have you been worried about being treated differently due to monkeypox? (Number of respondents: 736)	
Yes	39.8% (293)
No	37,5% (276)
I do not know	21.1% (155)
I do not want to answer	1.6% (12)

**Table 3:** Relationship between HIV status and knowledge about monkeypox, stigma, and vaccine acceptance.

	Comparison groups					p-value
People who are worried/very worried about the risk of acquiring monkeypox	HIV-positive		HIV-negative/unknown status			0.033
	43.6% (65/149)		34.1% (177/519)			
People who definitely or probably will get vaccinated if offered	HIV-positive		HIV-negative/unknown status			0.002
	80.6% (125/155)		67.6% (373/552)			
	On ART		Not on ART			0.046
	82.3% (121/147)		50.0% (4/8)			
People who think they will be treated differently due to monkeypox	HIV-positive		HIV-negative/unknown status			<0.01
	53.3% (81/152)		37.0% (203/548)			
	Age category in years					0.038
	18–24	25–34	35–44	45–54	55+	
	31.6% (49/155)	42.9% (140/326)	41.3% (71/172)	52.9% (27/51)	30.0% (6/20)	

## Footnotes

1 World Health Organization (WHO) (2023). Mpox (monkeypox) [online]. Website https://www.who.int/news-room/fact-sheets/detail/monkeypox [accessed 12 April 2023].

2 World Health Organization (WHO) (2023). Joint ECDC-WHO Regional Office for Europe Mpox Surveillance Bulletin: 04 April 2023 [online]. Website https://www.who.int/europe/publications/m/item/joint-ecdc-who-regional-office-for-europe-mpox-surveillance-bulletin--04-april-2023 [accessed 04 April 2023].

3 World Health Organization (WHO) (2023). Multi-country monkeypox outbreak: situation update [online]. Website https://www.who.int/emergencies/disease-outbreak-news/item/2022-DON393 [accessed 10 April 2023].

4 World Health Organization (WHO) (2022). Multi-country monkeypox outbreak: situation update [online]. Website https://www.who.int/emergencies/disease-outbreak-news/item/2022-DON396 [accessed 10 April 2023].

5 Anadolu Agency (AA) (2022). Türkiye reports 1st case of monkeypox [online]. Website https://www.aa.com.tr/en/turkiye/turkiye-reports-1st-case-of-monkeypox/2626527 [accessed 15 April 2023].

6 Centers for Disease Control and Prevention (CDC) (2022). 2022 Outbreak Cases and Data [online]. Website https://www.cdc.gov/poxvirus/mpox/response/2022/index.html [accessed 12 April 2023].

7 Centers for Disease Control and Prevention (CDC) (2022). 2022 Outbreak Cases and Data [online]. Website https://www.cdc.gov/poxvirus/mpox/response/2022/index.html [accessed 12 April 2023].

8 World Health Organization (WHO) (2023). Joint ECDC-WHO Regional Office for Europe Mpox Surveillance Bulletin: 04 April 2023 [online]. Website https://www.who.int/europe/publications/m/item/joint-ecdc-who-regional-office-for-europe-mpox-surveillance-bulletin--04-april-2023 [accessed 04 April 2023].

9 Centers for Disease Control and Prevention (CDC) (2022). 2022 Outbreak Cases and Data [online]. Website https://www.cdc.gov/poxvirus/mpox/response/2022/index.html [accessed 12 April 2023].

10 American Men’s Internet Survey (AMIS) (2022) Mpox Supplemental Survey [online]. Website https://emoryamis.org/ [accessed 15 Apr 2023].
